# Distributing Publicly-Funded Influenza Vaccine—Community Pharmacies’ Perspectives on Acquiring Vaccines from Public Health and from Private Distributors in Ontario, Canada

**DOI:** 10.3390/pharmacy9020094

**Published:** 2021-04-24

**Authors:** Joseph Fonseca, Richard Violette, Sherilyn K. D. Houle, Nancy M. Waite

**Affiliations:** 1 School of Pharmacy, University of Waterloo, 10A Victoria St S, Kitchener, ON N2G 1C5, Canada; joseph.fonseca@uwaterloo.ca (J.F.); richard.violette@griffithuni.edu.au (R.V.); sherilyn.houle@uwaterloo.ca (S.K.D.H.); 2 School of Human Services and Social Work, Griffith University, 68 University Dr, Meadowbrook, QLD 4131, Australia

**Keywords:** influenza vaccine, supply and distribution, community pharmacy services, public health, vaccination

## Abstract

Objectives: To explore community pharmacies’ experience with two models of distribution for publicly-funded influenza vaccines in Ontario, Canada—one being publicly-managed (2015–2016 influenza season) and one involving private pharmaceutical distributors (2016–2017 season). Methods: Online surveys were distributed to community pharmacies across Ontario during the 2015–2016 and 2016–2017 influenza seasons with sampling proportional to Ontario Public Health Unit catchment populations. Quantitative data were analyzed descriptively and inferentially and qualitative data were summarized for additional context. Results: Order fulfillment appeared more responsive with the addition of private distributors in 2016–2017, as more pharmacies reported shorter order fulfillment times (*p* < 0.01); however, pharmacies reported significantly more days with zero on-hand inventory in 2016–2017 (*p* < 0.01), as well as more instances of patients being turned away due to vaccine unavailability (*p* < 0.05). In both seasons, a similar proportion of pharmacies reported slower order fulfillment and limited order quantities early in the season. Improved availability early in the season when patient demand is highest, more vaccines in a pre-filled syringe format, and better communication from distributors on product availability dates were recommended in qualitative responses. Conclusions: Introducing private distributors for the management and fulfillment of pharmacies’ orders for the publicly funded influenza vaccine appeared to have mixed results. While key concerns surrounding the frequency, responsiveness, and method of delivery were addressed by this change, challenges remain—in particular, acquiring sufficient vaccine early in the season to meet patient demand. As pharmacies become more prominent as vaccination sites, there are several opportunities to ensure that patient demand is met in this setting.

## 1. Introduction

Historically, publicly funded vaccinations in Canada were completely managed within the public sector, from ordering and distribution through administration by public health departments or physician offices. In recent years, multiple Canadian jurisdictions have outsourced components of their publicly funded vaccination strategies to the private sector, including parts of vaccine distribution and administration. For example, most jurisdictions have introduced regulations that grant pharmacists vaccine administration authority, and while there is considerable heterogeneity, there is a trend towards public payers remunerating pharmacists for their administration of publicly funded vaccinations [1,2,3]. The earliest and the most widespread public–private partnership (PPP) taking advantage of pharmacists as immunizers in Canada has involved integrating their services with annual influenza vaccination programs [4]. The first Canadian province to build such a relationship with pharmacists was Alberta in 2007, with several other provinces making pharmacists part of their publicly-funded influenza programs in subsequent years [4]. As a result of these relationships, pharmacies are now the primary site for influenza vaccinations across Canada, and in Ontario—the most populous jurisdiction—the involvement of pharmacists as influenza immunizers since 2012 has led to improved influenza vaccination rates and a predicted societal net positive financial impact [4,5,6].

When pharmacists were introduced into Ontario’s Universal Influenza Immunization Program (UIIP) in 2012, the decision was made to use the existing government-managed influenza vaccine supply chain—coordinated and executed by the Ontario Government Pharmaceutical and Medical Supply Services (OGPMSS). From 2012 to the end of the 2015–2016 influenza season, all healthcare providers with Toronto postal codes (Ontario’s largest center, population ~3 million) had their vaccines delivered by the OGPMSS; in areas outside of the specified Toronto postal codes, vaccines were transferred to regional Public Health Units (PHU) throughout the province, and healthcare providers coordinated pick-up or delivery of their vaccine orders from their PHU. This system was revised prior to the 2016–2017 influenza season, with major changes affecting pharmacies outside of Toronto. Under the new system, all healthcare providers, including pharmacists, within Toronto continue to receive their vaccines directly from the OGPMSS, but for community pharmacies located outside of specified Toronto postal codes, distribution of influenza vaccines is now coordinated through approved pharmacy distributors. Private pharmaceutical distributors delivering publicly-funded vaccines to community pharmacists represents the PPP of interest, as there appears to be limited work in this space, particularly from the perspective of an end-user (i.e., immunizers, including physicians, pharmacists, etc.). It appears that most work exploring the outsourcing of vaccine delivery logistics has thus far focused on optimizing national health systems; within this work, there appears to be no Canadian or North American accounts of outsourcing in this way [7,8,9,10,11]. Further, the work that has explored immunizers’ experience with the logistics of vaccine delivery has not had an opportunity to explore the differences between public or privately-run delivery strategies—instead, largely focusing on satisfaction with an existing system or challenges faced in a particular season [12,13]. Finally, there appears to have been no published work aimed at understanding pharmacists’ experience with vaccine delivery strategies to date. The work presented here has an opportunity to contribute novel insight given its unique timing and perspective.

This work aims to explore the impact of introducing private pharmaceutical distributors as third-party logistics providers to community pharmacies in the Ontario influenza vaccine supply chain. Of particular interest were community pharmacies’ perspectives on: whether the supply of influenza vaccines was adequate to meet patient demand; how the mechanics of vaccine distribution may impact patient access to vaccinations; and opportunities for continued improvement in vaccine distribution for this setting. This data are particularly pertinent today, as there remain important knowledge gaps that could inform the redesign of vaccine supply chains—particularly those that involve PPPs. Previous work has acknowledged that clinician input could enhance the structure and function of PPPs [14], and understanding front-line pharmacists’ experience with these systems could inform the roll-out of pandemic vaccines—including COVID vaccines—that pharmacists may provide to the public.

## 2. Methods

### 2.1. Study Design

This project involved two cross-sectional online surveys, delivered via Qualtrics survey software (Qualtrics, Provo, UT, USA). The first survey took place before the changes to Ontario’s influenza vaccine distribution strategy and captured the 2015–2016 influenza season; this survey was distributed in February 2016 and received responses through the end of March 2016. The second survey took place after the aforementioned changes to distribution were implemented for the 2016–2017 influenza season; this iteration was distributed in March 2017 and continued until May 2017 (Data collection took place later in the 2016–2017 season as there was a longer influenza season [15,16] and the intention was for respondents to provide a complete picture of their 2016–2017 experience). Study protocol and methods received ethics approval from the University of Waterloo Office of Research Ethics (ORE #30749).

### 2.2. Data Collection

Survey questions were developed by pharmacy practice researchers, with input from practicing community pharmacists and pharmacy advocacy organizations. Questions focused on the logistics of ordering and receiving the seasonal influenza vaccine, including differences among the various vaccine formats (e.g., pre-filled syringes, multi-dose vials, and intranasal formulations), and the performance of the vaccine distribution system being used in that season. Open-ended survey questions were included to provide a more in-depth understanding of the quantitative data, particularly in the areas of pharmacists’ overall perceptions of the distribution system, areas for improvement and distributor performance, such as why order fulfillment times were delayed or partial orders were received.

### 2.3. Sampling

Participant recruitment was based on the Ontario College of Pharmacists’ publicly available register of community pharmacies and their staff. Sites that have accreditation as community pharmacies but operate in another setting (e.g., hospital pharmacies or Canadian Forces’ pharmacies) were excluded. Pharmacies were categorized by Ontario PHU (*n* = 36) using the University of Waterloo Geospatial Centre resources. Sampling of pharmacies was then performed by first selecting the number of pharmacies to sample within each PHU proportional to its catchment (population per PHU/provincial population * number of community pharmacies in Ontario), and then selecting pharmacies randomly within each PHU. The aims of this approach were to ensure proportional representation of pharmacist respondents relative to the Ontario population, and to ensure representation from each PHU in the sample (i.e., Toronto is served by one PHU and represents 20% of the population, and thus a proportionate sample of pharmacists were asked to complete the survey). Selected pharmacies were screened using phone calls to determine if the pharmacy administered influenza vaccines as part of the UIIP and to identify the individual responsible for ordering the vaccine for their pharmacy; these individuals were asked to complete the survey through a Qualtrics link emailed directly to them. The same pharmacies who were sent the survey in the 2015/2016 influenza survey were re-surveyed in the 2016/2017 influenza season; however, data was not matched year-over-year as no identifying data were collected to ensure anonymity. In addition, the authors were unaware that a change would be made to the vaccine distribution system when they designed the 2015–2016 survey (thus data that could be used to match participants in future iterations were not collected in the 2015–2016 survey). In 2016–2017, it became clear that geographic information was important for understanding how changes to the distribution system affected the experience of pharmacies outside of Toronto postal codes; therefore, the first 3 digits of participants’ postal codes (i.e., Forward Sortation Area (FSA)) were collected in the 2016–2017 iteration of the survey. The use of internet protocol (IP) addresses captured by Qualtrics to match or geolocate respondents was explored; however, would require several assumptions, including that participants responded from a static IP address, rather than the more common dynamic IP addresses, using the same device at the same location in each year. Moreover, a protocol for excluding participants using a virtual private network (VPN) was not used, because survey links were emailed directly to registered Ontario pharmacists who had been screened over the phone. The procedures used to manage this unpaired data are described below.

### 2.4. Data Analysis

Data were not paired by participants or pharmacies across the two cross-sectional surveys. As mentioned, pharmacies with specific Toronto-area postal codes received their vaccines from OGPMSS in both 2015–2016 and 2016–2017. For the purpose of this analysis, all participants with these Toronto-area postal codes were removed from the 2016–2017 dataset (*n* = 9, Toronto participants removed from 2016 to 2017), because their distributor model had not changed from the year prior, but remained in the 2015–2016 dataset, because they had a shared experience with all other pharmacies in that season.

Quantitative data were analyzed using SAS Release 3.8 (SAS Institute Inc., Cary, NC, USA). Almost all quantitative data were collected as categorical counts; the exception to this was a continuous numerical response to the question, “On average, how long did it take between when an order was placed and when it was available for pick-up or delivered?”, where data were binned as part of cleaning. Therefore, chi-squared tests of significance were used for all conclusions provided in this paper.

Qualitative data from open-ended questions were relatively concise and limited to <50 words per question. A summary of participants’ remarks was prepared; the interpretation of these remarks was verified by a second reader and the two readers reached an agreement on any discrepancies in the summary of remarks. Sentiments that were shared among several participants and that provide context for quantitative data or further understanding of pharmacists’ experiences are presented and discussed.

## 3. Results

### 3.1. Participating Pharmacies and Vaccination Workflow

Characteristics of the participating pharmacies are shown in Table 1. Survey response rates were 45.2% and 31.4% in 2015–2016 and 2016–2017, respectively. The majority of participants in both surveys worked in pharmacy chains, banners, or franchises. Nearly all pharmacies accepted walk-in requests for influenza vaccination in both influenza seasons with about one-third also offering the service during clinics or by appointment. A minority of pharmacies provided >500 influenza vaccinations in either 2015–2016 (33.8%) or 2016–2017 (36.7%).

### 3.2. Ordering Logistics

Table 2 presents key indicators of participating pharmacies’ experience with ordering and fulfilment. There was a statistically significant difference in ordering method between years (*p* < 0.01); ordering by fax dominated (91.0%) in 2015–2016, and online orders grew from 9.0% to 91.4% year-over-year. There was a statistically significant difference (*p* < 0.01) in the reported average time to order fulfilment between years, with a higher proportion of respondents in 2016–2017 stating that their orders were ready for pick-up or were delivered within 1–3 days compared to 2015–2016. However, in both seasons, >30% of respondents indicated that order fulfilment was slower earlier in the season compared to the remainder of the season. In the 2016–2017 season, a higher percentage of respondents reported that the distributor limited their order quantities both in early and late season compared to 2015–2016.

### 3.3. Patient Impact

Table 3 presents key indicators about the impact of vaccine ordering and fulfilment on patient care. Respondents in the 2016–2017 season reported significantly more instances where they were unable to provide patients with an influenza vaccination due to the vaccine being unavailable (*p* < 0.05). These 2016–2017 respondents also reported significantly more days with zero influenza vaccine inventory (*p* < 0.01). In both seasons, approximately 40% of participating pharmacies indicated that they ran out of the specific vaccine formats requested by patients at one point.

### 3.4. Qualitative Results

The results of key qualitative responses are presented in Table 4. Identifiable themes within each year are noted, and a year-over-year comparison is provided.

## 4. Discussion

Several significant improvements were seen following the introduction of pharmacy distributor-managed ordering and delivery of the publicly funded influenza vaccine; however, there are remaining challenges with important implications for when, and if, patients are vaccinated. For example, despite improvements in order fulfillment time with distributor-based vaccine management, limits on order quantities were more pronounced, and there were more pharmacies that reported instances of having no vaccine on-hand when patients requested it than under the public health distribution model. With the aim of vaccinating as many people as possible under a universal immunization program, efforts should be made to facilitate consistent product availability and stable pharmacy inventories, particularly at the start of the influenza season when public demand is highest, and pharmacies are running influenza clinics. To our knowledge, this is the first paper to examine healthcare providers’ experiences across two models of vaccine distribution—one being publicly-led and one being privately-managed.

To ensure representativeness, a novel sampling approach was utilized to identify the number of pharmacies from each PHU to be sampled in proportion to that region’s population and number of pharmacies. In both seasons, the majority of participating pharmacies were chains, banners, or franchises (62.9% and 59.3% in 2015–2016 and 2016–2017, respectively), which aligns with earlier surveys of Ontario community pharmacists on topics related to vaccines and immunization [17,18]. Differences in business models are not expected to have had a significant impact on pharmacies’ experiences with vaccine distribution from PHUs; however, in 2016–2017, there is the potential that corporate (chain, banner, franchise) relationships with private distributors could have influenced the distribution experience. Geographic differences were not analyzed, although they may also affect distribution experience.

In the 2015–2016 public-health managed distribution system, pharmacies felt they were spending a disproportionate amount of time on influenza vaccine inventory management compared to their other pharmaceutical inventory; this system relied heavily on faxed orders, that—in most cases—were fulfilled once per week, and required pharmacies to coordinate pick-up of vaccines from depots. In 2015–2016, pharmacies felt that more frequent order fulfillment, faster turnaround times on orders placed, and vaccine delivery were opportunities for improvement. The introduction of pharmacy distributor-managed ordering and delivery of the influenza vaccine appears to have brought considerable efficiencies from pharmacies’ perspective.

The statistically significant shift in ordering method between seasons—where online ordering effectively replaced fax ordering as the primary method—can be attributed to pharmacy distributors already having online ordering infrastructure that, in many cases, was integrated with pharmacy management software(s). This paperless ordering also appears likely to have contributed to pharmacies’ perception of increased system “efficiency”, as it aligned with how pharmacies were placing orders for all other products. It is not surprising that pharmacy distributors could offer more responsive order processing and fulfillment for pharmacies—seen with the increase in pharmacies reporting 1 to 3-day lead times—as delivering to pharmacies was previously not a core task of PHUs. 

Despite improvement in overall order processing and fulfillment, respondents in both seasons indicated that orders placed early in a season were typically slower to arrive than those placed later, and that quantities on these early orders were often limited by the distributor, whether that be PHUs or pharmacy distributors. Pharmacies believed that quantity limits were based on prior year vaccine ordering patterns and usage, as well as preferential allocation of vaccines to physician offices. We were unable to identify evidence that these strategies are mandated at the provincial level; however, we recognize these may be part of internal communications between the Ministry of Health and PHUs/distributors. The past and current vaccine order forms used by Ontario PHUs require a count of on-hand vaccine inventory, ostensibly to limit stockpiling; this may offer an alternative explanation for ordering limits experienced beyond the initial order [19]. Similar barriers related to orders being limited or late were identified by physicians in a 2005–2006 survey in the United States, although other studies of immunizers’ experience with the vaccine supply chain remain very limited [12].

Importantly, pharmacies in both seasons indicated that these bottlenecks had a negative impact on pharmacy workflow, but perhaps more importantly, had both direct and indirect implications for patients’ access to vaccination. For example, in both seasons, pharmacies discussed that significant time was spent resolving order issues with the distributor and explaining shortages to patients—both of which presented challenges to the pharmacy workflow. As mentioned previously, more pharmacies reported stock-outs when receiving vaccines from pharmacy distributors, and this likely contributed to more reports of patients being turned away. Some respondents indicated that they coordinated patient wait lists for those who wanted to receive the vaccine when the pharmacy was re-stocked, but this was another time-intensive activity for the pharmacy. The management of wait lists was also complicated by pharmacies not having estimated fulfillment dates for their orders. These challenges are not new nor unique to the pharmacy profession, as parallel frustrations have been expressed among physicians more than a decade ago, including the need to spend more time and effort obtaining vaccines, and a lack of communication from distributors surrounding the status of the supply chain [12].

### Addressing Remaining Challenges

While resolving early-season supply issues is complicated, communication between distributors and pharmacies can be improved by informing pharmacies of their initial allotment of vaccine, as opposed to asking pharmacies to submit order quantities which are then restricted. Anticipated delivery dates should be provided to assist pharmacies with managing wait lists and patient queries. Anticipated delivery dates may also allow pharmacies to avoid stock-outs while minimizing on-hand inventory, as it clearly indicates when just-in-time ordering (i.e., with next day delivery) is not possible. Allowing for a greater supply of vaccine doses early in the season or allowing the distribution of partial packages may also help meet patient demand while reducing wastage. It is notable that pharmacies explained that they were forced to over-order late in the influenza season as demand for the vaccine waned, because distributors only sold vaccines in multiples of the pack size (e.g., 1 pack = 8 vaccine doses). It is unknown how many unused doses were ultimately returned at the end of the influenza season, that could have been offered to individuals impacted by shortages elsewhere. Finally, a more drastic redesign of the current system could involve a shift towards vendor-managed inventory (VMI)—where distributors receive on-hand inventory data from healthcare facilities and determine replenishing shipment quantities based on this data instead of from purchase orders. VMI systems have proven beneficial in some countries that also outsourced logistics of publicly-funded vaccine distribution activities [20].

## 5. Conclusions

Moving forward, vaccine supply chain redesigns are opportunities for research at the intersections of economics, epidemiology, and social sciences. There remains a lack of real-world accounts surrounding these redesigns, and even fewer that consider the impact on immunizers as end consumers in these systems. With pharmacists’ growing role as immunizers in Canada and globally, the processes and frameworks for designing these types of outsourcing arrangements and best practices for implementing them appear to be underdeveloped and may lack considerations for pharmacies as sites of immunization. Finally, these findings and recommendations are particularly relevant in an age where pandemic responsiveness within vaccine supply chains and immunization capacity are at the fore. Existing pharmaceutical distribution infrastructure will need to be leveraged in creative ways to achieve rapid and widespread rollout of any pandemic-relevant vaccine or medication and appreciating the challenges that pharmacies face in acquiring and managing their inventories, depending on the distributor and systems in place, can inform how we design pandemic response strategies.

## Figures and Tables

**Table 1:** Characteristics of participating pharmacies.

	2015/2016 n (%)	2016/2017 n (%)
**Type of pharmacy**	140 (100)	81 (100)
Chain ^1^, Banner, or Franchise	88 (62.9)	48 (59.3)
Mass Merchandiser/Grocery Store	26 (18.6)	19 (23.5)
Independent	26 (18.6)	14 (17.3)
**How does the pharmacy organize flu vaccinations (select all that apply)**	144 (100)	81 (100)
Appointment	50 (34.7)	29 (35.8)
Flu clinics	42 (29.2)	18 (22.2)
Walk-in Patients	144 (100)	78 (96.3)
**Average daily prescription volume**	130 (100)	78 (100)
</=50	12 (9.2)	6 (7.7)
51–150	56 (43.1)	29 (37.2)
151–250	37 (28.5)	20 (25.6)
251–350	8 (6.2)	9 (11.5)
351–450	13 (10.0)	3 (3.8)
451 or more	4 (3.1)	11 (14.1)
**How many vaccines did you administer this flu season?**	145 (100)	71 (100)
Zero	10 (6.9)	0 (0.0)
1 to 249	58 (40.0)	33 (41.8)
250 to 499	28 (19.3)	17 (21.5)
500 to 749	19 (13.1)	13 (16.5)
750 to 999	12 (8.3)	6 (7.6)
1000 to 1249	9 (6.2)	10 (12.7)
1250 to 1499	2 (1.4)	0 (0.0)
1500 to 1749	3 (2.1)	0 (0.0)
1749 to 1999	1 (0.7)	0 (0.0)
>/= 2000	1 (0.7)	0 (0.0)

^1^ “chain” refers to any 6 or more stores with the same owner(s).

**Table 2:** Ordering and fulfillment experience.

	2015–2016 n (%)	2016–2017 n (%)
**Ordering method (select all that apply)**	145 (100)	81 (100)
Online orders	13 (9.0)	74 (91.4)
Telephone	10 (6.9)	3 (3.7)
Fax	132 (91.0)	20 (24.7)
Email	3 (2.1)	1 (1.2)
**Did the supplier limit the quantity that could be ordered early in the flu season?**	147 (100)	81 (100)
Yes	99 (67.3)	67 (82.7)
No	48 (32.7)	14 (17.3)
**Did the supplier limit the quantity that could be ordered later in the flu season?**	146 (100)	81 (100)
Yes	32 (21.9)	35 (43.2)
No	114 (78.1)	46 (56.8)
**On average, how long did it take between when an order was placed and when it was available for pick-up or delivered?**	141 (100)	81 (100)
1 to 3 days	73 (51.8)	66 (81.5)
4 to 7 days	58 (41.1)	9 (11.1)
8 to 14 days	7 (5.0)	5 (6.2)
15 days or more	3 (2.1)	1 (1.2)
**Did order fulfillment time change throughout the flu season (referencing above, select all that apply)?**	147 (100)	82 (100)
Slower earlier in the flu season	45 (30.6)	27 (32.9)
Slow in the middle of the flu season	4 (2.7)	3 (3.7)
Slower at the end of the flu season	14 (9.5)	13 (15.9)
Not applicable	84 (57.1)	39 (47.6)

**Table 3:** Impact of vaccine ordering and fulfilment on patient access to vaccines.

	2015–2016 n (%)	2016–2017 n (%)
**Were there instances where you could not give influenza vaccine to patients who wanted it due to vaccine not being available?**	143 (100)	81 (100)
Yes	58 (40.6)	47 (58.0)
No	85 (59.4)	34 (42.0)
**How many days during the flu season were you out of vaccine?**	123 (100)	52 (100)
No days	60 (48.8)	6 (11.5)
1 to 3 days	16 (13.0)	11 (21.2)
4 to 7 days	26 (21.1)	12 (23.1)
8 to 14 days	14 (11.4)	15 (28.8)
15 days or more	7 (5.7)	8 (15.4)
**Were there instances where you were out of the format of vaccine patients requested (e.g., FluMist^®^, preservative free)**	142 (100)	78 (100)
Yes	62 (43.7)	32 (41.0)
No	80 (56.3)	46 (59.0)

**Table 4:** Pharmacists’ written responses to questions surrounding their vaccine distribution experience in subsequent influenza seasons.

Did the Vaccine Distribution Experience Impact Daily Functioning of the Pharmacy?		
2015–2016	2016–2017	Similarities and Differences
Time Requirements Additional time was dedicated specifically for vaccine ordering from PHUs because it followed a separate process than other drug orders. Time was also spent communicating directly with PHUs to resolve ordering and fulfillment issues Time required to pick-up the vaccines from depots was burdensome, particularly for pharmacies that were not near a PHU depot	Increased Efficiency Deliveries in this season were a notable efficiency for many Supply Chain Frustrations and New Time Requirements Some vaccine formats were frequently unavailable through their suppliers Time was spent communicating with distributors directly to resolve ordering issues Time was spent explaining shortages to patients, and in some cases coordinating wait-lists for yet-to-be delivered vaccines Additional time was spent ordering vaccines than other drug products, because pharmacies were searching all available products to see which formats had inventory for next-day delivery	Reasons as to why the vaccine ordering process was cumbersome changed; some problems were resolved, and new challenges presented themselves In both years, fulfillment was a challenge and pharmacies noted having to contact their distributor, whether that was the PHU or a private distributor
**What reasons were provided for initial and early-season order quantities being limited?**		
**2015–2016**	2016–2017	Similarities and Differences
Quantities were limited by PHUs Pharmacists indicated that quantity limits were based on use in the previous season and ensuring all pharmacies received some vaccine inventory	Quantities were limited by distributors Pharmacists indicated that quantity limits were based on use in the previous season and ensuring all pharmacies received some vaccine inventory	In both seasons, pharmacists indicated similar rationale and understanding as to how initial order and early-season order quantities were allocated or limited
**What improvements to the current influenza vaccine distribution process could be made?**		
**2015–2016**	2016–2017	Similarities and Differences
Desire to have orders fulfilled more than once per week Desire for vaccine delivery to the pharmacy Desire to have vaccines available earlier in the season Desire for more vaccines in a pre-filled syringe format to enable pharmacy workflow efficiencies Faster turnaround on order submission and fulfillment required to better match demand	Desire to have vaccines available earlier in the season Desire for more vaccines in a pre-filled syringe format to enable pharmacy workflow efficiencies Desire to have suppliers provide approximate or specific dates as to when vaccine formats will be back in stock and ready for delivery when back-orders occur Wasted supply or over-ordering due to distributors requiring orders in multiples of pack sizes; pharmacies could not order single doses or return leftover doses	In both seasons, pharmacists indicated that distributing the vaccine earlier in the season and providing more vaccines in a pre-filled syringe format would improve their experience as UIIP providers. The desire for more frequent order fulfillment, faster turnaround times upon ordering, and delivery appear to be resolved in the 2016–2017 season, without a single comment related to these themes. Requests for more specific dates of availability, and the option to order in quantities that were not multiples of pack sizes or to return un-used vaccine doses from an opened pack were new themes in 2016–2017.

## Data Availability

Data are available from the authors upon request.
